# Are social causes so different from all other causes? A comment on Sander Greenland

**DOI:** 10.1186/1742-7622-2-4

**Published:** 2005-05-24

**Authors:** Ezra Susser, Sharon Schwartz

**Affiliations:** 1 Department of Epidemiology, Columbia University, Mailman School of Public Health, 722 West 168th Street, New York, NY 10032, USA; 2 Epidemiology of Brain Disorders at the New York State Psychiatric Institute, NY, USA; 3 Associate Professor of Clinical Epidemiology, Columbia University, Mailman School of Public Health, 722 West 168th Street, New York, NY 10032, USA

## 

Sander Greenland's elegant paper [6] raises deep questions about the way in which we choose and evaluate public health actions. We agree with the main thrust of his argument with respect to public health policy. We have some concerns, however, about his treatment of social causes, and on this point we focus our critique.

Greenland proposes a counterfactual definition of a cause which is framed in terms of alternative actions with different potential outcomes. He suggests that, under this framework, social conditions – such as socioeconomic status, sex, or race – present a quandary for causal inference. They cannot be considered as causes unless they can be reframed in terms of alternative actions. Greenland's approach to this problem is, thankfully, not to dismiss social causes, but rather to "identify potential causes within ordinary events". He suggests that we consider alternative actions to change social conditions, and their different potential outcomes.

We do not believe it is warranted to single out the identification of social causes or characteristics as posing an especially severe problem for causal inference. Even under this framework of alternative actions, similar problems pertain to all kinds of exposures in observational studies. It is important to acknowledge this similarity, because if social exposures are perceived as being the most problematic for studies of causation, investigations of these causes and their remediation may be put at a disadvantage.

First, in our identification of their causal effects, most of the exposures we study are more akin to conditions than actions. To infer a causal relationship for an exposure, we imagine that we could remove that exposure while "all other things remained equal", and compare the outcomes under the exposed and unexposed conditions. In other words, we compare what happened under the condition of exposure with what would have happened under the condition of no exposure with "all else held constant".^1 ^This is not truly equivalent to a comparison of the outcomes of alternative actions, such as removing or not removing the exposure. If we had actually removed the exposure, all other things would not have remained equal.

Most of the causes we study are similar to social causes in this respect. Consider the classic example of smoking cigarettes. If people were unable to smoke cigarettes, they might as a result drink more alcohol, have more episodes of depression, or gain more weight. All other things would not remain equal. Thus, when we construct a counterfactual that compares smoking with no smoking, we are not truly comparing the potential outcomes of alternative actions. We are constrained to comparing the outcomes under two alternative conditions, one of which is necessarily counterfactual.

Second, most of the exposures we examine can be seen as the consequence of a previous action, and as a possible mediator of its effect on health. As noted earlier, Greenland suggests that we could reframe social causes (e.g. years of education) as potential outcomes of previous alternative actions (e.g. better schools to improve educational outcomes), which may in turn improve health. But with equal legitimacy, most other exposures could be reframed in the same way. We could reframe physical activity as a potential outcome of previous alternative actions to increase physical activity, which may in turn improve health. For all these exposures, we continually strive to better understand both the antecedents which lead to the exposures, and the biological mechanisms which connect them to disease outcomes.

Third, although some conditions turn out to be more manipulable than others, we do not often know beforehand which ones they will be. A researcher's judgment about what conditions are and are not manipulable tends to be influenced more by values than by scientific empirical data. We certainly have no empirical data to support the view that social causes are generally less manipulable than others. On first impression, cigarette smoking may appear to be a readily manipulable action. But it has turned out to be extremely difficult to reduce the smoking epidemic worldwide over the past half century. While cigarette consumption per person has declined in some high income countries over the past few decades, the global impact on health continues to accumulate, with a predicted rise in smoking-related illness and death in low and middle income countries, where the vast majority of the world's more than one billion smokers now live [1,2]. On the other hand, raising levels of education, which may at first seem a more difficult task, has actually been achieved throughout much of the globe over the same period. With regard to "fixed" characteristics such as sex, we seek to modify their relationship to health and disease, by manipulating biological and social experiences alike.

Notwithstanding these differences, we concur with Greenland on his central point about the formulation of public health policy. The effects of public health actions and policies "do not correspond to simple cause removal" {editor, citation to Greenland}. Therefore, we should clearly differentiate two endeavors: the identification of causes and the evaluation of interventions to remove these causes.

Suppose we initiate a public health action to reduce smoking in a population. Whatever action that may be (e.g. banning the production and sale of cigarettes), it is not plausible to think that it could result in a population in which smoking cigarettes had been reduced while all other things remained equal. The population will change in its composition and historical time; the decline of the cigarette industry may lead to unemployment and consequent ill health in some regions, the opening of new markets for cigarettes in other areas, and so on.

Epidemiologic studies of causes provide crucial clues to the design of preventive interventions, but they do not provide good estimates of the impact of these interventions. Like Greenland, we advocate studies that directly compare the effects of alternative public health actions (one of which may be inaction). We also believe that, insofar as possible, these studies should compare the effects of alternative actions across multiple health domains rather than only a single domain.

Finally, we suggest that although epidemiologists most often study conditions rather than actions, counterfactual reasoning is applicable to our discipline. The inclusion of conditions as causes has a long tradition under counterfactual reasoning. In an early contribution to counterfactual reasoning about causation, the philosopher Mackie [3] argued that we must consider conditions as well as actions to be potential causes (chapter 2). In the field of psychology, Shadish et al. [4] adopt the counterfactual approach to defining causes, and explicitly state that the causes so defined include nonmanipulable as well as manipulable events. From epidemiology, we quote Rothman and Greenland: "We can define a cause of a specific disease as an antecedent event, *condition, or characteristic *that was necessary for the occurrence of the disease at the moment it occurred, given that other conditions are fixed." [5] (p. 8, our italics).

## Note

As Greenland describes, when we study an exposure as a cause, our goal is to compare the outcomes of the same people in the exposed and the unexposed state. We cannot achieve this goal, but only approximate it. Thus we directly measure the outcomes under the exposed state, which did occur, but not under the unexposed state, which did not occur and is therefore counterfactual. We use the outcomes in another, unexposed group as a proxy for what would have happened to the exposed group had they not been exposed.
